# “Age independent, but person dependent”: a Swiss interview-based study on the meaning of good parenthood at an advanced parental age

**DOI:** 10.1186/s12910-025-01259-5

**Published:** 2025-07-11

**Authors:** Nathalie Bettina Neeser, Andrea Martani, Veerle Provoost, Guido Pennings, Bernice Simone Elger, Christian De Geyter, Nicolas Vulliemoz, Tenzin Wangmo

**Affiliations:** 1 Institute for Biomedical Ethics (IBMB), University of Basel, Bernoullistrasse 28, Basel, 4056 Switzerland; 2 Department of Philosophy and Moral Science, Bioethics Institute Ghent (BIG), Ghent University, Ghent, Belgium; 3 University Center of Legal Medicine, University of Geneva, Geneva, Switzerland; 4 Reproductive Medicine and Gynaecological Endocrinology (RME), University Hospital, University of Basel, Basel, Switzerland; 5 Centre de Procréation Médicalement Assistée (CPMA), Lausanne, Switzerland

**Keywords:** Advanced parental age, Parenthood, Child welfare, Ageing, Ethics, Qualitative methods

## Abstract

**Background:**

Child welfare is one of the most important topics when it comes to parenting competence and the definition of good parenthood. This is widely discussed, especially in the context of treatment considerations for medically assisted reproduction (MAR) for patients of Advanced Parental Age (APA, here defined as 40 years and older). The aim of this study is to contribute to the exploration of how different stakeholders in this context envision the concept of good parenthood.

**Methods:**

An explorative semi-structured interview study was conducted with a total of 15 healthcare providers, 12 aspiring APA-parents, 21 APA-parents and 20 adult offspring of APA-parents.

**Results:**

After thematic analysis, results show that although the connecting focal point among participant groups is reproductive age, participants consistently emphasized that APA is not a determining factor to define a “good” parent. Instead, we identified three themes representing participants’ views on this topic: (i) the difficulties in defining good parenthood; (ii) the family structure and context as inherent to parenting quality; and (iii) good parents as conscious adapters.

**Conclusions:**

Participants expressed their views that good parenthood depends on the personality of the parent, rather than on one single characteristic of a parent, such as age. Our results challenge the focus on a singular parental characteristic in safeguarding the welfare of future children and therefore also the role currently attributed to parental age in decisions about access to MAR.

**Supplementary Information:**

The online version contains supplementary material available at 10.1186/s12910-025-01259-5.

## Background

Child welfare is one of the most important and widely debated topics in relation to treatment considerations in medically assisted reproduction (MAR) [2, 38, 43]. Healthcare providers are continuously confronted with ethical questions regarding access to MAR [4] and have to balance the aspiring parents’ autonomy, whilst at the same time protecting the best interest of the potential child(ren) [23]. In recent decades, there have been attempts to determine what child welfare entails as a criterion to grant access to MAR. According to Pennings, this can be interpreted in three different ways: (i) the maximum welfare principle, meaning that a child should only be brought into the world under ideal circumstances; (ii) the minimum threshold principle, meaning that a child can be brought into the world if it has at least a life worth living; and (iii) the reasonable welfare principle, which does not aim at a perfectly happy child but rather a reasonably happy child [37]. However, it is difficult to implement any of these standards in practice when it comes to granting (or denying) access to MAR. It is more common for specific features of the aspiring parent(s) to be used as criteria to assess whether they will be good parents, such as the ability to provide a stable family environment or exhibiting stable mental health [23].

Advanced parental age (APA) is one of the most common parental characteristics used to restrict access to MAR based on concerns regarding child welfare [39]. While becoming parents later in life raises doubts about the capacity of raising a child [20], APA is becoming increasingly common. In 2002 in Switzerland, only 2.5 per cent of all births were from women 40 years and older, whereas in 2022 this percentage had increased to 6.1 per cent of all births [16]. The use of MAR among APA-mothers, defined as those 40 + is, as in 2019, around one fifth (19 per cent) of all IVF treatments in Europe [44]. In Switzerland, the percentage of women using IVF treatments at 40 years or older, was one quarter (24.5 per cent) in 2022 [6]. Although statistics about paternal age are limited, it can nevertheless be pointed out that in 2023, 20.2 per cent of fathers in Switzerland were ≥ 40, and more than half of all fathers (51.9 per cent) were ≥ 35 when their child was born [7]. Additionally, it is known that in 2022, men in Switzerland were on average 39.6 years old when using IVF [6]. Although the proportion of advanced age fathers may seem excessive, high paternal ages are not unprecedented [49].

When it comes to the experiences of APA parents, the majority of studies focus on APA mothers, showing that they experience an increased sense of stability, are more confident and are generally more satisfied about being mothers at APA [11, 18, 19, 30]. However, existing literature also shows that APA mothers describe their experiences at APA as physically challenging [11, 27, 30] and report having experienced social stigma being labelled as “old mothers” [10, 18, 27, 30]. When it comes to APA fathers, a recent study reports that APA fathers distance themselves from the perception that they are “too old” to be parents and that some experienced social stigma – similar to APA mothers – leading to various coping strategies [41].

Conceiving later in life often requires MAR due to age-related decline in fertility, but many countries have set maximum age limits for aspiring APA-parents to access MAR [45, 50]. This is often justified by concerns that conception at an APA may negatively impact the welfare of the children. Swiss law, for example, allows access to MAR only to parents “who, on the basis of their age and personal circumstances, are likely to be able to care for and bring up the child to majority.” (our translation) [5]:251). This rule was justified, in the preparatory works of lawmakers, by reference to the lively ethical and societal debate about the potential discouragement or prohibition of APA parenthood [ 9, 22, 39]. However, it is difficult to define what makes a good parent and there is no consensus on this point [4, 39]. It is also unclear whether there is a connection between APA, parental capacity and the welfare of the child, and thus whether discouraging oreven prohibiting APA in the context of MAR is justified [40].

With this study, we aim to explore how different stakeholders involved in MAR or with direct experience of APA envision the concept of good parenthood. We conducted interviews with adult children of APA-parents, aspiring APA-parents currently undergoing MAR, APA-parents who recently had children, and healthcare providers working in the context of MAR. In these interviews, we asked them about their views on what makes a good parent and elicited their opinions on the potential connection between parental age, parental capacity and child welfare.

## Methods

### Study design

This study was conducted as part of the A-PAGE project, which focuses on family building at APA. The goal of the project is to examine how age influences family dynamics and the broader implications on moral, social, and legal aspects of family building. The project includes various components among which the exploratory semi-structured interviews discussed in this study. This study was approved by the ethics commission Nordwest- und Zentralschweiz (EKNZ, BASEC-Nr. 2021–01429). We followed the COREQ checklist for reporting qualitative research [46].

### Sampling and data collection

Our goal was to recruit participants from four different groups: (i) adult offspring who had at least one parent who was 40 years or older when the participants were born; (ii) aspiring APA-parents who did not yet become parents but who were in the process of undergoing MAR-treatment; (iii) APA-parents who had successfully undergone MAR-treatment and who had already become parents at 40 years or older; and (iv) healthcare providers working with APA-parents using MAR. Although there is no universal definition of APA, a systematic review of the literature found that previous studies have defined advanced *maternal* age as everything from ≥ 31 to ≥ 50 years and advanced *paternal* age as everything from ≥ 40 to ≥ 55 years [34]. For our study, we chose to define APA as ≥ 40 years at birth of the child, because this cut-off point is often used for both mothers [3] and fathers [12]. With global life expectancy extending from 72.8 years in 2018 to 77.2 years in 2050 [47], and even reaching 82.2 years for men and 85.8 years for women in the context of Switzerland [8], defining APA as ≥ 40 might seem odd at first glance. However, the *reproductive* span is entirely different from the *life* span, specifically in the case of women. In fact, Nabhan and colleagues [33] stated that women’s fertile period starts around the age of 13 to 15 years and ends with menopause around the age of 45 to 50 years. However, women experience fertility decline with advancing age and the median age at which they have their last child lies around ∼40 to 41 years when conceiving without medically assisted reproduction [14]. Although men’s reproductive span is much longer, we decided to define one cut-off point for APA instead of defining advanced *maternal* and advanced *paternal* age, making ≥ 40 a sensible choice.

The study was designed as an explorative qualitative study. To reach maximum variation in the sample, we used both purposive [15] and snowball sampling [36, 48]. Several recruitment strategies were used, depending on the four groups of participants: (i) the offspring group was recruited through personal contacts of the first author (*n* = 17); the advertisement of the study in an online forum (*n* = 2); and the referral of other participants (*n* = 1). Meanwhile, the (aspiring APA-parents and the healthcare providers, were mostly recruited with the help of study collaborators, working at fertility clinics in both the German and French speaking part of Switzerland (*n* = 34), followed by a midwifery-newsletter (*n* = 6), personal contacts of the first author (*n* = 5), the institute’s website (*n* = 2), and participant referral (*n* = 1). We recruited a total of 20 offspring with at least one APA-parent, 12 aspiring parents of APA or with an APA-partner, 21 APA-parents, and 15 healthcare providers, which led us to a total sample of 68 study participants. Interviews with adult offspring with only one APA-parent strongly focused on the experiences of participants with their APA-parent instead of with their non-APA-parent. In detail, this meant that the interview guide was structured in a way that in one part, the focus lied on the mother and in another on the father, with a specific focus on the parent that was 40 year or older, if the offspring had only one APA-parent and one that was younger at the time of their birth. Interviews with aspiring parents who were not of APA themselves, strongly focused on their views and their meaning-making of their partner’s APA. We did not ask adult offspring *how* they were conceived – naturally or with the help of medically assisted reproduction – as we invited the participants to share their stories in a way that allowed them to include what was meaningful to them. Furthermore, this question would potentially not have been answerable, as not all parents choose to disclose their (in-)fertility journey to their offspring. As for the APA-parent group, study collaborators specifically approached advanced age mothers, as they worked especially close with them during their treatment, leading to more women than men in our sample. No participants who initially expressed interest in participating changed their mind. The most important demographic characteristics of the study participants are presented in Table 1.

**Table 1:** Demographic characteristics of the different participant groups

Participant group	Characteristics	Variable	*N*
Offspring with at least one APA-parent (*N* = 20)	Gender	Female	13
		Male	7
	Age	18–21 years	4
		22–25 years	3
		26–29 years	7
		30–33 years	5
		34–37 years	1
	Age of mother at birth	28–39 years	12
		40–44 years	8
	Age of father at birth	28–39 years	3
		40–59 years	17
Aspiring parents undergoing MAR treatment (*N* = 12)	Gender	Female	8
		Male	4
	Age	36–39 (with a 40 + partner)	3
		40–43	9
APA-parents (*N* = 21)	Gender	Female	15
		Male	6
	Age at birth of their child(ren)^a^	Up to 35 years	3
		36–39 years	6
		40–43 years	20
		44–47 years	3
		48 years and older	4
Healthcare providers (*N* = 15)	Gender	Female	13
		Male	2
	Profession	Physician	4
		Nurse	3
		Midwife	4
		Others	4

^a^The 21 participants included in the APA-parent group had a total of 36 children. For parents to take part in the study, it was a requirement that they had at least one child (not necessarily the first) at 40 years or older

### Interviews

Semi-structured interviews were carried out between March 2022 and November 2023. Most interviews were carried out in a one-on-one setting (*n* = 52), while 16 participants preferred to participate in the study together with their partner. Depending on their preferences, participants were either interviewed in person (*n* = 36) or online (*n* = 32). As the topic of the study was a very sensitive and potentially challenging one, we asked participants to choose in which setting – with their partner or alone; in person or in an online setting – they felt most comfortable to participate in the study. In the interviews conducted with a partner present, participants often shared and compared experiences, values and views, and thereby deepened the conversation. All interviews were carried out by the first author, who introduced herself as a PhD student interested in parenthood at APA. The first author was trained in qualitative methods and experienced in conducting interviews with different study groups on a variety of topics. About one week prior to the interview, study participants were provided with all necessary study information and an informed consent form. After reviewing the study information and answering any questions, participants provided written informed consent. Additionally, offspring, aspiring parents and parents were asked to fill out a demographic data form after their participation in the study.

Four tailored semi-structured interview guides were designed for the participant groups (see supplementary file). Participants of all groups were asked about what makes, in their opinion, a good parent and if age had anything to do with it. The interview guides for offspring and healthcare providers were pilot-tested before recruitment started. No repeat interviews were conducted. Of the 60 interviews (with 68 participants), 42 were conducted in (Swiss) German, 10 in French, and 8 in English. Interviews ranged between 37 and 158 min, with an average of 82 min. Field notes were taken during and after the interviews. The interviews were recorded and transcribed verbatim. The first author conducted the interviews and sought to explore and understand the participants’ perceptions, interpretations, and experiences related to APA. To enhance reflexivity, the first author maintained a research diary to document and reflect on any potential biases and personal experiences encountered during the recruitment and interview stages. This diary was later referenced during the data analysis process, and notable reflections were discussed collaboratively with the co-authors. All identifiable information, such as names, places and dates were pseudonymised. Transcripts were not returned to participants for their comments and they were not asked to provide feedback on the findings.

### Analysis

The first author analysed the data using applied thematic analysis [21] and engaged in inductive coding using the qualitative analysis software MAXQDA. She engaged in an initial analysis and invited co-authors (AM, VP, GP, BE, TW) to participate in an interdisciplinary collaborative auditing meeting [42] during which the first author presented a selection of quotes, illustrating the thematic structure of the analysis. Co-authors were tasked to challenge the interpretation and the grouping of the codes into the presented themes. They participated in a discussion of the presented quotes leading to adaptations and refinement of the thematic structure. During the auditing meeting, the diverse backgrounds of the co-authors – encompassing social anthropology, gender studies, bioethics, gerontology, legal analysis, medicine, and philosophy – enabled an interdisciplinary approach that strengthened and deepened the thematic structure. In a second auditing round, co-authors were provided with a draft of the manuscript and were invited to provide their feedback on it, which refined the final thematic structure.

## Results

**Fig. 1:**
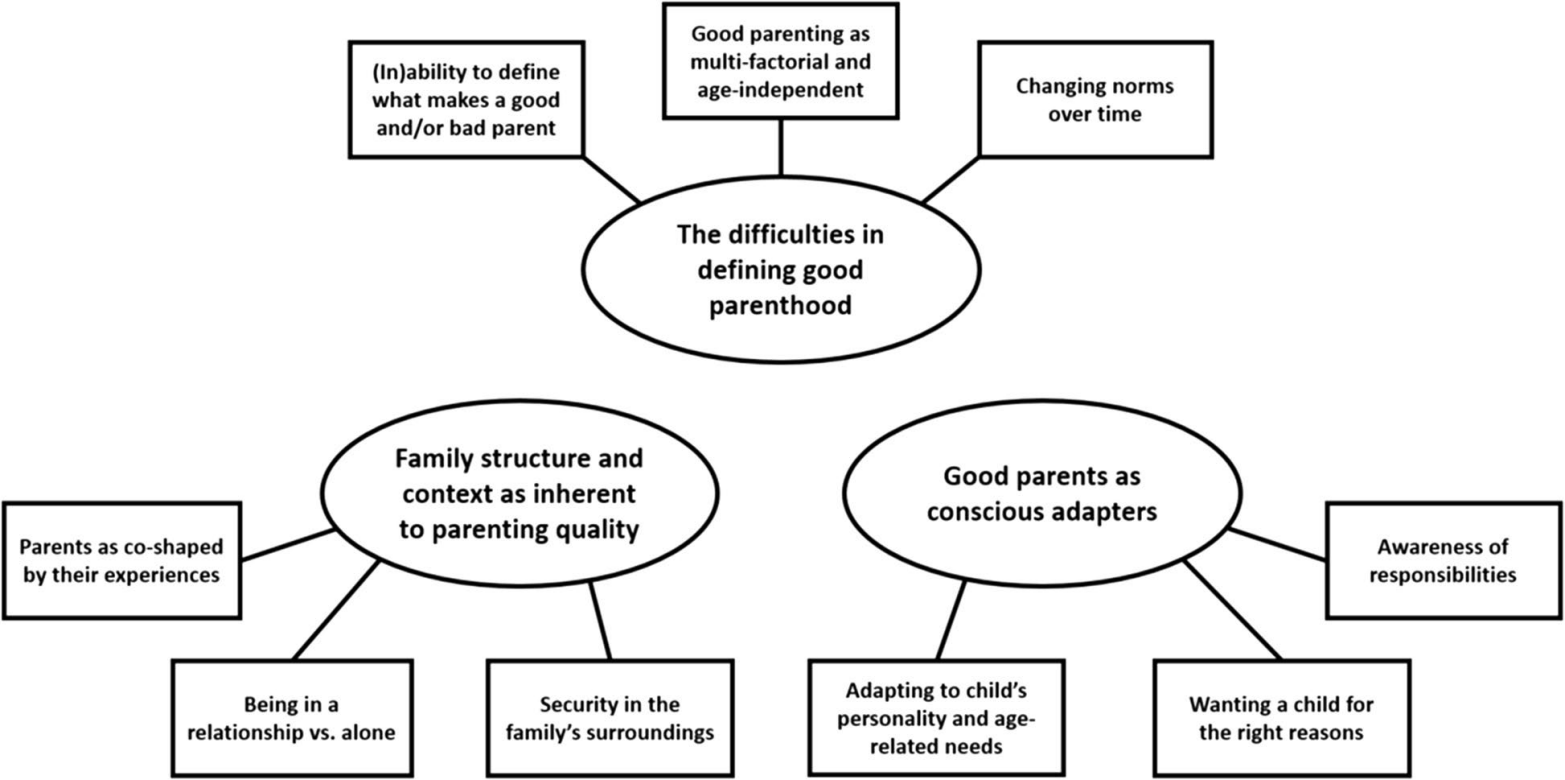
Thematic structure to define a good parent

In the following, we present three descriptive themes, representing the views of the stakeholder groups included in this study. The first theme describes the difficulty participants voiced in defining which characteristics make a good parent, and their inability and unwillingness to come up with a potential definition. The second theme relays the importance participants attributed to the overall family structure and the family’s surroundings, and not just the parent(s) themselves, in what contributes to creating a good parenthood experience for children and their welfare. The third theme presents the significance of parenting adaptability, depending on different individual factors, and the awareness of the parental responsibility one has for their child (see Fig. 1). In the following, we discuss each theme with quotes from study participants. To increase readability and still be as detailed as possible, we use abbreviations: (i) offspring will be abbreviated as Off01 to Off20; (ii) aspiring parents as AP01 to AP12; (iii) parents as P01 to P21; and (iv) healthcare providers as HCP01 to HCP15.

### The difficulties in defining good parenthood

When participants were asked how they would define the term good in terms of being a parent, many participants were initially baffled by the question, as evident by the following sub-themes.

#### (In)ability to define what makes a good and/or bad parent

Several participants started by saying that it was too difficult to define a good/bad parent, followed by questioning whether they even had the authority and/or the qualification to provide a generalizable answer in the first place. A parent (P11) explained: “*I find that a bit presumptuous to define that for all parents*” and a healthcare provider (HCP06) specified: “*Everyone has to decide that for themselves. […] You can’t define that from the outside [of the family]*”. While some participants struggled to come up with generalizable definitions for *all* parents, others struggled particularly because they explained parenthood to be a private, rather than a public issue and they did not perceive themselves to have the authority nor the expertise to judge others. Yet, several participants tried to investigate the nuances involved in the definition. In order to do so, a healthcare provider (HCP07), came up with a comparison to romantic relationships.

*You cannot tell from the outside [of the family]. I think that’s another one of those situations, similar to: Which relationship is good and which isn’t? Because from the outside, there are families that you see a photo of and then you think: That’s the perfect family. But then you get closer and maybe you realize: It’s not all that great.* – HCP07

Other participants questioned who would generally be authorized to decide whether someone is a good parent or not. One offspring even reflected on a potential tool to assess good and bad parenthood and concluded that it would be much easier to define the latter.

*Every now and then, I really wish there was a test for people who want to become parents, to make sure that they are suitable. I am serious, because with some parents, I just think: How can you be a mum or a dad? And then there’s also that, well, what’s good is more difficult to define than what’s bad, I think.* – Off06

The participant (Off06) described that she had experienced first-hand what bad parenthood looked like. She grew up with an abusive parent and explained that there were certain characteristics that clearly defined a bad parent. Several other participants voiced the same conviction that defining bad parenthood is easier than good parenthood.

#### Good parenting as multi-factorial and age-independent

Many participants described good parenthood as a multi-factorial issue. A healthcare provider (HCP02) clearly expressed this by saying that “*All aspects are relevant, aren’t they?*” and another (HCP07) insisted that “*There is not one single characteristic that says: They are good parents.*” While some participants only made these general statements, others detailed that the capacity to be a good parent has nothing to do with someone’s age. A participant from the offspring group (Off14) explained: “*I think you can be young and be a good mum or a good dad and you can be old and be a good mum or a good dad. Or you can be bad.*”. Similarly, a participant from the healthcare provider group (HCP04) brought the discussion to an individual level and away from one single determinant and said: “*I think that’s age-independent. It’s person-dependent.*”

#### Changing norms over time

Finally, several participants reflected on the question by focusing on how parenthood and the societal views attached to it have changed over time. That is, parental characteristics and identity traits might be good in the present, even though they might have been characterized differently in the past. Amongst several participants who expressed these views, two stood out (HCP06 and P08). A healthcare provider stated that heteronormativity was still strongly anchored in society and explained: “*They [a man and a woman] are married and now they have a child. That still counts as good enough, but that’s just no longer up to date*” (HCP06). In a similar tone, but considering a different issue, a parent expressed that there exists a double standard of what is expected of a “good” parent, depending on their gender. While a good father may be described in a certain way, the description may not apply to a mother.

*I always have big issues with my mum, who still lived in the old pattern. And I also see this old role model in my environment: My friends are all around my age, they’ve all studied, they all have good jobs, they all work. And they all have mothers like my mum, who was at home 100 per cent of the time. […] And there are a lot of discussions with our mothers. […] Because women are simply expected to be completely free of needs when they have children. Simply because of their love for the child they are so happy that they no longer have any other needs. And that’s not true.* – P08

### Family structure and context as inherent to parenting quality

Despite initially refusing to clearly define good parenthood, most participants then mentioned some characteristics that define whether someone qualifies as a good parent. One such characteristic was the family structure and context which, according to most participants, was inherent to parenting quality.

#### Parents as co-shaped by their experiences

According to several participants, the experiences of parents while growing up and before having children, play a role in defining their parental capabilities and suitability to parent. A healthcare provider (HCP14), explained having had a very difficult childhood and initially not wanting to be a mother: “*I couldn’t imagine being a mother, given what I went through as a child. In my mind, it made sense not to have any [children]*”. However, this participant stated that she eventually decided to do everything in her power not to repeat the past and wished not to miss out on the experience of having children herself. It is in a similar tone that another participant described her opinion about the past influencing the present and the future.

*It also depends on the relationship we’ve had with our parents. If we had a good relationship, we’re likely to have a good relationship with our children. Or if we didn’t have the right relationship, we’ll do everything we can to try and have the right relationship with our own children. Finally, we mustn’t repeat what has already happened in the past.* – P14

Most participants concluded that past experiences – both positive and negative – were important in shaping someone as a potential parent, but that these experiences were ultimately not the defining factor in the assessment of someone’s current parental capacity. In fact, having negative past experiences was also seen as an opportunity to do better, while positive past experiences were seen as something to learn from and be inspired by. However, in both cases it was seen as important to not simply sit back, but to actively work on one’s parental capacities. Moreover, for most participants, other aspects of the parental background played similarly important roles. One of the participants (P11) made this clear as she reflected on the role the parents’ age plays in the definition of their capabilities, but finally concluded that other aspects weighted much heavier: “*Age is one thing, but social status or income or where you stand in the social class is something else. If you have parents who are alcoholics or drug addicts…*”. Finally, a factor that was also often mentioned was financial security, with participants mentioning that *“perhaps older parents have different resources than younger parents”* (P20). However, this was not characterized as a determining factor for good parenting, but rather as something that makes life easier, as other participants made clear.

*I personally don’t find the financial aspect* that *[the participant’s emphasis] important. It’s certainly* also *[the participant’s emphasis] important, in that you are definitely better off than when you, for example, have a child during your studies.* – Off04

#### Being in a relationship vs. alone

Although being in a well-functioning relationship was not claimed as a necessity, it was nevertheless seen as helpful to achieve good parenthood, as pointed out by a parent (P09).

*I think you can only judge that [whether someone is a good parent or not] by the quality of the relationship with the child. And also by the relationship between the parents themselves. If you have a good relationship [with your partner], you’re likely to be a good parent.* – P09

However, what did not matter to most participants was the couple’s sexuality. A healthcare provider (HCP10) said: “*Who’s good enough to be a parent? Maybe a couple, be it man-man, or woman-woman, or woman-man, let’s say. But I think a couple is an advantage*”. However, the participant pointed out that this was not a requirement, which was confirmed by another participant as well.

*The partnership is of course also important. If the partner is right, you can look after the children well together. And otherwise, everyone has to look after the children on their own. But you can definitely look after the children well, even if you live separately.* – HCP02

One of our participants was a single mother by choice. She explained at length that she had intensively thought about being alone on her parenting journey, but that she ultimately came to the conclusion that it was better to do it alone than with someone who was not suitable. This corresponds to the opinions of most of our participants, as being in a suitable partnership was seen as an advantage, while being in a relationship with someone who was not up to the challenge to be a parent was not.

#### Security in the family’s surroundings

According to some participants, an important element for children’s welfare is not the presence of a parent per se, but rather of anyone who takes on the challenge of fulfilling a caregiving role: “*It does not necessarily have to be a parent, but there has to be a reference person. I would describe it as a reliability, security, closeness, for them to be there*” (Off03). Although only a handful of participants argued that there was no specific need for a parent, but rather for a parental figure to take on what was characterized as parental responsibilities, several others discussed the importance of the social environment in which children grow up in determining whether the children’s welfare is maintained. A parent (P06) explained: “*I think I would define good parents as parents that manage to provide their children with an environment in which they feel protected, loved, supported, recognized, valued, loved*”. Several participants did not specify whether this had to be an environment created by parents or rather just by people interested in the well-being of the child.

### Good parents as conscious adapters

After the participants’ initial bafflement about the question and its complexity, many participants eventually discussed good parenthood by reflecting on what competences individuals have to bring to the table and which requirements they need to meet to successfully engage in this role.

#### Adapting to child’s personality and age-related needs

Several participants noted a feature that parents should possess in order to ensure the welfare of a child: the capacity to adapt to the age and the personality of the child and to the needs arising from it. A participant from the parent group (P01) said that being a good parent generally means responding to your child’s needs “*in a phase-appropriate way*”, e.g. depending on the age of the child. Other participants were more specific and pointed to the fact that with each new life stage, one has to assess what the child and the relationship to the child needs specifically, making parenthood a job that requires tact and sensitivity. As an example, a participant from the offspring group (Off06) clarified how a parent might have to evaluate the relationship repeatedly throughout the child’s life and specifically during the child’s puberty: *“There are certainly phases where you realize that your child just doesn’t feel like it anymore. Then you can push a little. But then there’s also the question: when do you push and how hard?”.* Additionally, participants also underlined that it is not only about the child’s age, but also about the child’s personality. A healthcare provider (HCP07) explained: “*It also partly depends on the children. Not all children from the same couple are equally demanding, equally easy*”. Another offspring (Off15) specified: “*Not all children are so easy to love. Because some are a bit exhausting, like all of us human beings. And you can’t be friends with everyone and you can’t be a good parent to everyone*”. It becomes clear, that being a good parent ultimately not only depends on the parent in question, nor on the age of the child, but in fact on the interactions between parent and child.

*It always takes two. No matter how good a mother or father you are, if your child rebels, it won’t do you any good. If your child is stubborn or has their own opinion, then you can use any method or be as good as you like. At some point, it just won’t work anymore. That’s why I believe that whether someone, whether a mother or a father, is good is an interplay between the child and the parents. It takes both. – *Off02

Even though some of the participants clearly identified the importance of an interplay between parents and their children, they were nevertheless clear that the child is the vulnerable party in the relationship. In fact, several of our participants acknowledged the importance that the parents identified and adapted to their child’s needs and that the child was provided with all necessary means to choose their own path in life. A participant from the parent group (P11) illustrated: “*I think the most important thing is that the child can go through life independently and that it has a good backpack with certain tools on its shoulders*”. Finally, some participants also reflected on the parent’s age when it came to the parent’s ability to identify what a child needs in a given life stage or situation.

*Maybe I need to be 45 to be able to identify my child’s needs. Because before that, I couldn’t even identify my own. And to be able to identify a need, not to be emotional, not to be intellectual, but to be at the level of need: that’s complex. And there are people who do this very intuitively, maybe they’re even able to do it at the age of 16. And for others, it takes longer. But really, I think that’s it. A child is a little being in the making. It’s immature, it needs to grow, it has needs, it is like a plant that needs to be in soil. And I need to know what that soil is, and as a parent, I need to do my best to put the child in that good soil. And for me, a good parent is just that. It’s not a parent who always does everything right, it’s a parent who knows how to identify their child’s needs and respond to them.* – HCP15

In the end, most participants came to the conclusion that parenting was a delicate process which has to be re-negotiated repeatedly over time.

#### Wanting a child for the right reasons

Additionally, a point that was repeatedly mentioned as a given was that parental competence required a conscious decision as well as the ‘right’ motivation. In fact, the consciousness involved in the decision-making to become a parent was repeatedly pointed out. An offspring (Off08) for example said: “*I believe that if you do it [become a parent], you have to make a conscious decision and then really accept the consequences*”. This was not the only thing that was identified as a must. For most of our participants it was clear: the child has to be wanted for its own sake not for the parent’s:

*Sometimes I have the impression that some people just have a child because they want to be pregnant or they just want to have a baby. But it’s not just about your needs, but that you’re aware of what you’re doing and what kind of commitment you’re making for the rest of your life. That you have this desire and can stand behind it 100 per cent.* – Off15

The participant emphasized that you do not qualify as a good parent if you decide to have a child simply to fulfil your own needs, but that you have to want a child because you want to be there for them. Finally, being ready and making a conscious decision to be a parent was something that was linked to age by many of our participants. Even though most participants were certain that one could be a good parent at any age and that age did not have anything to do with the necessary parental competence, many participants nonetheless pointed out that increasing age made it more likely that someone was in fact, making a conscious decision to take on the commitment to be a parent.

#### Awareness of responsibilities

Most participants voiced the importance of continuously remaining aware of the responsibilities that the decision to become parents brings along. An aspiring parent (AP07) pragmatically stated: “*A good parent is certainly a parent who is aware of his or her role and duty*”. Additionally, many participants voiced how important it was to first properly think about the journey they would be embarking on and not take one’s parental role lightly. A healthcare provider (HCP05) said: *“It’s important to really think about: What kind of responsibility am I taking on? It’s not just cute and sweet and I’ve reproduced, but what does it mean to have a child?”.* While most participants talked about the responsibilities that come with being a parent, some participants did not use this specific wording, but rather talked about the consequences of becoming and being a parent. Additionally, one of the most often highlighted characteristic that makes a good parent was the awareness of the responsibility to be there for the child. One of the healthcare providers (HCP01) mentioned his own parents during the interview and that they were always there for him and were reliable. He explained: “*I have complete trust in them and I always know that I can count on them. I think that’s something very important*”. And even though, dedicating time to the child and being present was described as sounding easily achievable, this aspect has layers to it, as one of the parents (P02) pointed out: “*For me personally, it’s important that I’m present. Not just physically, but that I’m really there. Also in the future for the children. That they know: you can always come home, I’m interested in what’s going on with you*”. And while one might not always succeeds to be there for the child when they need their parent, participants pointed out that the bottom line was that a child can think of their parent as a reliable person in their lives.

## Discussion

In this study, we analysed how different stakeholders, namely offspring of APA-parents, aspiring APA-parents, APA-parents and healthcare providers working with APA-parents using MAR, define “good parenthood” and what characteristics they attribute to good parents. We identified their difficulties in coming up with a definition and at the same time, we explored their many views of what makes a good parent. What stood out when engaging with the experiences and perspectives of the participants was that the age of a parent was not a central element influencing their views on parental competences and the qualification of someone as a good/bad parent. This is in contrast to practices in the context of MAR, where parental capacity is often discussed and questioned based on the advanced age of the parents in question [39]. This has many concrete implications, as demonstrated by a case from 2011 in Italy, when a Court removed a one-year old child from its parents (they conceived through MAR at ages 57 and 70) due to – amongst other things – considerations about their APA as impeding them to care for the child [22].

Most European countries have set different maximum age limits for aspiring APA-parents to access MAR [45, 50]. Switzerland is one of the very few countries in Europe without explicit chronological age limits for MAR [45]. In fact, the Swiss Federal Act on MAR only mentions age in one single instance, stating: *“[Assisted reproductive techniques] may only be used in couples […] who, on the basis of their age and personal circumstances, are likely to be able to care for and bring up the child until it reaches the age of majority”* [17]. In practice, this is often implemented so that access to MAR for aspiring fathers above 60 years is granted only if they provide a certificate of good health, whereas for aspiring mothers there are informal limits within each clinic at around 43 years of age – given that oocyte donation is not allowed [28]. Nevertheless, if a couple can conceive without MAR, the mother – and especially the father – can be older.

Some of our participants stated their belief that reproductive choices are private issues and voiced their uneasiness in defining in absolute terms what specific characteristics a good parent should possess. While for fertile adults reproduction and the choices whether/when to have children largely remain private issues, for infertile adults this is not the case. The fact that they have to rely on MAR to fulfil their child wish means that external elements (e.g. laws on MAR access, assessments by MAR professionals etc.) will co-determine their reproductive choices. This distinction between fertile and infertile aspiring parents can be defined as questionable and potentially unjust. Klitzman [23] demonstrates the difference in the assessment of fertile and infertile adults by reminding that fertile adults might freely have children regardless of their parenting capacities, as they are able to fulfil their child wish without external interference. For infertile adults, on the contrary, the fulfilment of their child wish requires the support of healthcare providers, which often entails a potentially arbitrary assessment of their parental capacities as future parents [23, 29]. In the UK, the Human Fertilisation and Embryology Act from 1990 tasks healthcare providers facilitating MAR to perform an assessment based on the concept of the welfare of the child [25]. This means, that a woman “*shall not be provided with [infertility] treatment services unless account has been taken of the welfare of any child who may be born as a result of the treatment*” [25]. It is not surprising that a legal situation like this means that healthcare providers feel they have to perform some sort of assessment to account for the welfare of the (future) child. It is however not clear, what exactly the healthcare providers assessing this, should aim for. This is coherent with our results, as our participants both expressed their uneasiness in assessing what characteristics might make for a good parent and stated that in the end, one cannot pinpoint it to one single characteristic. Rather, it is an interplay of different characteristics, and depends on the child in question. It is therefore not surprising that healthcare providers express being challenged when they have to perform such an assessment [23, 29].

Even when one of the standards to evaluate child welfare (the maximum welfare principle, the minimum threshold principle, and the reasonable welfare principle [37]) has been selected, hands-on parental characteristics are needed in order to assess child welfare in practice. The existing literature reports how individuals experience being APA parents, for example mothers feeling more confident, more stable and satisfied [11, 18, 19, 30] while both mothers and fathers also report experiencing social stigma due to their age [10, 18, 27, 41]. However, it remains to be discussed how these experiences might influence the parental capacity of APA parents. Nevertheless, in the past, groups of people with characteristics deviating from the parental norm, such as single aspiring parents, same-sex aspiring parents or trans aspiring parents [1, 13, 24] have been questioned in their parental abilities and were unfairly treated as a result. Even though in our interviews, participants did not mention their views on transgender individuals as parents, some of our participants voiced their opinions on same-sex couples and on single parents. That is, they thought that being in a relationship was considered an advantage, it was however not a necessity and the parental capacity of single parents was also not discarded. Regarding same-sex couples, the participants stated that the sexuality of parents was negligible when it came to their parental capacities. In fact, other aspects such as the ability to adapt to the child’s needs played a much bigger role in our participants’ opinions. Participants also provided their views on parental norms changing over time, reflecting on their perceptions how parental capacity was still anchored as a heteronormative and gendered issue. In sum, participants seemed convinced that the parental capacity and the potential definition of someone as a good parent was not something that could be pinpointed to one single characteristic of a person, such as their sexuality or their (advanced) age for that matter. In the participants’ views, a good parent was defined from a multitude of characteristics that are interacting with one another.

Finally, participants felt that it was much easier to come up with characteristics for a bad parent than for a good one. This finding corresponds to previous research findings. Aspects that have been shown to negatively influence the well-being of children are: being raised by a parent who has experienced child abuse themselves [26, 31], and being raised by a parent with a previous criminal conviction of child abuse [32]. However, even though these aspects have been shown to negatively impact parental capacities, our participants did not see them as absolute disqualifying factors for being a good parent. They agreed that, for example, for parents affected by abuse during their childhood, they may have to work through their own traumas, but they also felt that these parents still had the potential to become a good parent despite not having experienced good parenthood themselves. There are other characteristics that have been shown to negatively influence parenthood, such as the parents’ socioeconomic resources, their gender, their partnership status, their minority background and whether they belong to a sexual minority [35]. Even though our participants commented on the socioeconomic resources, the gender, and the partnership status of parents to potentially be defined as good parents, they contradicted the importance of a singular factor in two ways. First, they mentioned that even though a singular characteristic might seem essential, such as having experienced abuse as a child, the willingness of a parent not to repeat the past was considered more meaningful. Second, none of our participants provided one single characteristic to define a good parent, but rather searched for a definition in the multiplicity of different characteristics.

Our study has some limitations. First of all, our sample is self-selected and it is possible that we predominantly recruited participants who had rather positive experiences with APA. In fact, none of our offspring or (aspiring) parent participants openly said, that they had specific negative experiences with APA which would lead them to, for example, do things differently if they had the chance. Additionally, not all of the interviewed offspring had two APA-parents, but in some cases only one, which might have influenced their perception of APA and the presented results. Furthermore, when it comes to the demographic information, we were unable to provide any information on the ages and the parental status of the interviewed healthcare providers. Even though this information might have been illustrative, we decided to focus on the experiences participants had in their specific professions and did not collect such data. Also, we have chosen a specific definition of APA, namely someone who is 40 years and older when becoming a parent. In the literature, there is no clear-cut definition of APA and there are many different definitions to be found [34]. Finally, this study has a few minor methodological limitations. Participants took part in the study in different contexts (in person or online; alone or with their partner) which could have led to differences in what they disclosed, for example when their aspiring APA-partner was present. As for the strengths of this study, our qualitative approach in engaging with the growing phenomenon of APA-parents and their children allowed us to contribute to the debate around the assessment of parental capacity when it comes to MAR. Moreover, including four different stakeholder groups all connected in the focal point of advanced reproductive age has provided an exceptional sample and a rich collection of views and experiences to engage with in the analysis.

## Conclusions

With this study, our aim was to contribute to the exploration of how different stakeholders in the context of APA envision the concept of good parenthood. Not only did participants consistently emphasize that APA is not one of the determining factors of a good parent, but they stated that good parenthood depends on the person a parent is, rather than on one single characteristic of a parent, such as age. Our results therefore challenge the focus on a singular characteristic in safeguarding the welfare of (future) children and hence also the role currently attributed to parental age in decisions about access to MAR.

## Supplementary Information

Supplementary Material 1.

## Data Availability

The data that support the findings of this study are available from the authors upon reasonable request.

## Abbreviations

APA: Advanced Parental Age
AP01 – AP12: Interview participant group: Aspiring parents 01 – 12
EKNZ: Ethikkommission Nordwest- und Zentralschweiz
HCP01 – HCP15: Interview participant group: Healthcare providers 01 – 15
IVF: In-Vitro Fertilization
MAR: Medically Assisted Reproduction
Off01 – Off20: Interview participant group: Offspring 01 – 20
P01 – P21: Interview participant group: Parents 01 – 21
