# Supplementary material for: “Age independent, but person dependent”: a Swiss interview-based study on the meaning of good parenthood at an advanced parental age
Source: BMC Med Ethics. 2025 Jul 11;26:95. doi: 10.1186/s12910-025-01259-5 (PMC12247432; doi:10.1186/s12910-025-01259-5)
Supplement: Supplementary file 1 — Supplementary Material 1. [file 12910_2025_1259_MOESM1_ESM.docx]

**Supplementary file: Interview guides for the project “Family building at advanced parental age: an interdisciplinary approach”**

**Interview guide healthcare providers**

| **Part of the interview guide** | **Interview questions** |
| --- | --- |
| Potential follow-up questions/questions to keep the conversation going or to go into more depth:   - What does that mean exactly? Can you describe this in more detail? - Do you have an example so I can imagine this more concretely? - I don’t fully understand can you explain this a little more? - How was that for you? How did you feel at the time / what were you thinking? - Is there anything else? What else? And further? And then? - What do you mean by that? How do you mean that? How did it continue? | |
| Experiences in relation to reproductive technology | - I would like to start with a general question about your professional experience. Can you give me an overview of your professional background, especially in relation to reproductive technology? - Can you tell me a little more about your experience regarding treatments using reproductive technology with couples, of which at least one of them is over the age of 40? - What is the biggest challenge in the treatment of couples who use fertility treatments at an advanced age for you? And what is the most rewarding / gratifying thing about it?   *The following questions focus specifically on couples seeking fertility treatments at an advanced age.*   - Have you ever encountered challenges when it came to **the communication** with patients? If so, when and how? How did you deal with them? - Have you ever encountered challenges or obstacles in providing **optimal care** to patients? If so, when, which ones and to what extent? How did you deal with them? - How does the decision-making process generally work? Who is usually involved in the process and are there certain factors that influence the decision for or against reproductive technology? - Do you see areas where the care, awareness, or policies on reproductive technology for parents who are 40 or older could be improved? If so, which ones?  *Follow-up question: And in what ways could these be improved, in your opinion?* - What recommendations and what advice would you give to someone in a similar position to yours who is just beginning their career in the context of reproductive technologies and parents at an advanced age? What do you think is particularly important to know and to consider when caring for this target group in this context? |
| Moral reasoning to gain a new understanding of advanced parental age as a basis for better and more appropriate policies and recommend-dations | *For the next few questions, let’s take a bit of a step away from your very focused professional experiences that you’ve told me about so far to your general opinions and views. I would be interested in knowing…*     - Do you think society has a different image of older parents than of younger ones? - In your opinion, does society think differently about parents (especially older parents) who have to resort to reproductive technology to have children (compared to people who became parents naturally and earlier in their lives)? - In your opinion, what influence do social and cultural factors have on couples who want to become parents at an advanced age with the help of reproductive technology?   *Follow-up question: Do you think these factors affect men and women in the same way?* - In your opinion, could society and / or politics do something better to address the needs of couples at an advanced age who want to become parents using reproductive technology? If so, what exactly? - For my next question, I will use this timeline (see end of interview guide). On this timeline you can see different arrows: “too early to have children”, “quite early for children but acceptable”, “ideal time for children”, “a little late for children but acceptable” and “too late to have children”. As you can see, all of the arrows are the same length, however, this does not necessarily mean that the time spans must also be the same length. I would very much like to know what you think about this.  So my first question is: When is it too early, quite early, quite late or too late for children? Can you give a specific age? It doesn’t have to be exact, but it can be a period of time, for example five or ten years. Can you give any reasons for your statements?  *Follow-up question: Are there differences between men and women?* - In a general way, what is your opinion of parents who have children via reproductive technology at an advanced age?  *Follow-up question: What are the advantages and disadvantages for (a) parents; (b) child(ren); (c) the family as a whole?*   *After having talked in detail about parenthood at an advanced age, I would now – as the last topic of our conversation – be interested in your opinion about parenthood and family in general.*   - How would you define the term “good (enough)” in relation to parenting / being a parent?  *Follow-up questions: Does being older influence the meaning of “good” in connection with “being a parent” in any way? – Or are other factors more determining and consequently “more important”?* |
| Conclusion | Thank you very much. We have now discussed a number of things. Is there anything that you would like to mention or add that has not come up in the conversation so far, but that you think is important? Thank you very much for your participation. |
| Timeline exercise |  |

**Interview guide Offspring**

| **Part of the interview guide** | **Interview questions** |
| --- | --- |
| Potential follow-up questions / questions to keep the conversation going or to go into more depth:   - What does that mean exactly? Can you describe this in more detail? - Do you have an example so I can imagine this more concretely? - I don’t fully understand can you explain this a little more? - How was that for you? How did you feel at the time / what were you thinking? - Is there anything else? What else? And further? And then? - What do you mean by that? How do you mean that? How did it continue? | |
| Experiences related to having parents of an advanced age during childhood | - Before we begin, it would be nice if you could tell me a little about your family. That way I know who we are talking about during the interview and can follow your stories better. - Perhaps we can first talk about your childhood. How was the relationship with your parents during that time?  *Follow-up questions:* - *How did you perceive your parents at that age?* - *If you have sometimes talked about your parents with friends or classmates, how did you feel during these conversations?* - *Was this about the age of your parents?* - Was there a specific moment when you realized that your parents were a bit «older» than those of your friends / classmates?  *Follow-up question: If yes, when and how was that exactly? How did you feel about it?* |
| Experiences related to having parents of an advanced age during adolescence and childhood | - Now let’s move on to the time of your youth. What was your relationship with your parents like during that time?   *Follow-up questions:*   - *How did you perceive your parents during that time?* - *Has your relationship with your parents changed compared to when you were a child?* - Do you think your parents’ age has affected your relationship and interactions with them in any way? Can you briefly explain why (not) and in what way exactly?  *Follow-up questions:* - *When you compare your relationship with your parents with that of your friends / classmates who have younger parents, do you see any differences?* - *Did it sometimes feel like your family situation was different? Or that you were treated differently?* - Now, I would appreciate it if you could give me three adjectives that describe your current relationship with your mother. You are welcome to calmly take some time to do this.   *Follow-up question: For what reason did you choose these words? Can you explain it to me in a little more detail?* - Next, I would appreciate it if you could give me three adjectives that describe your current relationship with your father. You are welcome to calmly take some time to do this.   *Follow-up question: For what reason did you choose these words? Can you explain this to me in a little more detail here as well?* - If we now turn to your adulthood: How has your relationship with your parents changed from your childhood, through your adolescence, to today’s adulthood? - If we now dare to look into the future: What do you think your relationship with your parents will be like in the near future, let’s say in 5-10 years?  *Follow-up question, if the person is 18-25 years old: What will it look like in 10-20 years?*   **Questions in case the interview participant is an only child:**   - You had mentioned earlier, when you were describing your family situation, that you are an only child. Could you tell me how you think this influenced or still influences your relationship with your parents?  *Follow-up question: What was this like as a child? As a teenager? In adulthood?* - What is your impression what consequences / effects if will have on the future of you and your parents that they had «only» you as their only child?  *Follow-up questions:* - *What might be advantages and disadvantages of being an only child of parents at an advanced age?* - *Do you ever think about caring for and/or looking after your parents as they age or, perhaps, talk to your parents about this or similar aspects of their aging?* - *If yes: Do you have the impression that you think about this because (1) you are an only child; (2) because your parents were already at an advanced age at the time of your birth; or (3) because of both of the mentioned aspects?* |
| Experiences and views on parenthood | - What does it mean to you personally to have “older” parents?  *Follow-up question: Are there advantages and/or disadvantages to having older parents?* - Do you think society has a different image of older parents than of younger ones?  *Follow-up question: How does this make you feel?* - In your opinion, what influence do societal and cultural factors have on couples’ desire to become parents only at an advanced age?  *Follow-up question: Do you think these factors affect men and women in the same way?* - Due to advancing age, some couples resort to reproductive medicine to fulfill their desire to have children. What are your thoughts and opinions regarding this topic?  *Follow-up question: Do you think society views couples who use reproductive medicine differently than couples who have children through natural means?* |
| Moral reasoning to gain a new understanding of parenting at advanced age as a basis for better and more appropriate policies and recommend-dations | - Have you ever thought about having children yourself?  *Follow-up question: Are there any personal or external factors that influence you in this decision (e.g. career, partner, etc.)?* - Is there in any case something in particular that you would do differently than your parents? Can you briefly explain why (not)? - How would you define the term “good (enough)” in relation to parenting / being a parent?  *Follow-up question: Does being older (when you think of your own parents, for example) influence the meaning of “good” in connection with “being a parent” in any way? – Or are other factors more determining and consequently “more important”?* - For my next question, I will use this timeline (see end of interview guide). On this timeline you can see different arrows: “too early to have children”, “quite early for children but acceptable”, “ideal time for children”, “a little late for children but acceptable” and “too late to have children”. As you can see, all of the arrows are the same length, however, this does not necessarily mean that the time spans must also be the same length. I would very much like to know what you think about this.  So my first question is: when is it too early, quite early, quite late or too late for children? Can you name a specific age? It doesn’t have to be exact, so it can be a period of time, for example five or ten years. (+Can you give reasons for your statements?)   *Follow-up questions:*   - *Are there differences between men and women?* - *Are there any advantages and / or disadvantages for (a) parents; (b) children; (c) family as a whole?*   We are already almost finished!   - For this last exercise, your answers to these following exercises do not have to match what you have said so far. I would like to get your first impression on these topics here. So it would be good if you answered as if we had just met.  So, I have now brought some statements (see end of interview guide) that are based on newspaper articles, social media posts and books. So they do not correspond to my own opinion. I will read you one statement after another and then give you the corresponding card. Please rate each statement whether you agree or disagree with it and place the card either in the “I agree” column or in the “I disagree” column. Please think out aloud why you are making which decision so that I can understand why you are choosing which option. |
| Conclusion | Thank you very much. We have now discussed a number of things. Is there anything that you would like to mention or add that has not come up in the conversation so far, but that you think is important? Thank you very much for your participation. |
| Timeline exercise |  |
| Statement exercise | I agree / I do *not* agree   \| 1. Parents should be able to take care of their child. \| 4. It is best for aspiring parents to start having children as early as possible. \| 7. Imagine a single parent with health problems. His or her child of 15 years can be expected to do something for that parent. \| \| --- \| --- \| --- \| \| 2. Parents should only be able to take care of their child until it has a certain age, after that they do not have to ‘care’ anymore. \| 5. When a parent needs care himself, his or her children can be expected to take care of that parent. \| 8. If a parent is not able to play sports with his or her child, then he or she is actually too old. \| \| 3. Age has nothing to do with how good or bad someone can raise a child. \| 6. Imagine a single parent with health problems. His or her child of 35 years can be expected to do something for that parent. \| 9. People who have children later in life have to make an effort to look good so that they do not embarrass their children. \| |

**Interview guide parents**

| **Part of the interview guide** | **Interview questions** |
| --- | --- |
| Potential follow-up questions/questions to keep the conversation going or to go into more depth:   - What does that mean exactly? Can you describe this in more detail? - Do you have an example so I can imagine this more concretely? - I don’t fully understand can you explain this a little more? - How was that for you? How did you feel at the time / what were you thinking? - Is there anything else? What else? And further? And then? - What do you mean by that? How do you mean that? How did it continue? | |
| Experiences in relation to reproductive technologies | - Before we begin, it would be nice if you could tell me a little bit about your family. That way I know who we are talking about during the interview and can follow your stories a little easier. - Could you tell me about the moment when you and your partner started thinking about having children? How was this? What were your thoughts and feelings about starting a family? - Could you tell me a little more about your attempts to get pregnant when you first developed a desire to have children?  *Follow-up questions:* - *How did you feel when you realized that it did not work out right away with getting pregnant?* - *Did you search for support or advice (from friends, family, doctors, etc.)?* - How did the decision-making process take place?  *Follow-up questions:* - *Have you talked to your partner, your family, your friends, your doctors?* - *Have you considered alternatives to these reproductive technology options (e.g. adoption)?* - *If yes: Was there anything about these alternatives that put you off, causing you to decide against them?* |
| Experiences with the treatment | - Could you tell me your experience with the treatment from beginning to end?  *Follow-up questions:* - *Did you feel supported during the process?* - *What role did your partner, your family, doctors / health care professionals play?* - *Were you afraid of possible stigmas?* - Do you have the impression that your age affected the treatment in any way? - Has the search for reproductive health care support affected your relationship with your partner? - Looking back, is there anything you would have done differently? |
| Views about parenthood | - At the time you started thinking about having a child, what did it mean to you to become a parent? - What does it mean to you now? - How did your family and your social environment (e.g. friends, work colleagues, etc.) react when they found out about your wish to have a child and/or about your pregnancy? - How do you experience being a parent? - How do you experience your own age in relation to being a parent?   *Follow-up questions:*   - *Are there any advantages or disadvantages to being a parent at your age?* - *What about advantages and disadvantages for (a) you as a parent; (b) your children; (c) the family as a whole?* - *In general, not just in connection to your own experiences: In your opinion, what are generally the advantages and disadvantages to parenting at an advanced parental age?* - *What are your thoughts with regard to the size of your family? Did your age at the time of your desire to have children influence the size of your family, respectively the number of your children in any way?* - *Would you describe yourself as a parent of advanced age?* - *At what age would you generally describe someone as a “parent of advanced age”?* - How would you describe the relationship with your child / children?  *Follow-up questions:* - *Do you think that your age has an impact on your relationship with your child / children?* - *Do you think that your relationship with your child will develop differently than if you had become parents earlier? In what way?* - *What do you think about how your relationship with your child / children will develop in the future, when your children are grown up and you yourself are already “older”?* - *If we now look one step further into the future: How do you think your age will affect your relationship with your grandchildren (if your child / children decides to become parents themselves)?* - Dou you think society sees you differently than younger parents?   *Follow-up questions:*   - *How does this show?* - *How do you feel about it and how do you react to it?* |
| Moral reasoning to gain a new understanding of advanced parental age as a basis for better and more appropriate policies and recommen-dations | - Did society, culture, and / or the health care system (or other external factors) play a role in you “only” becoming a parent at this age? - In your opinion, what do you think is the influence of these external factors, like social or cultural factors, on couples who become parents at an advanced age?  *Follow-up questions:* - *Are there differences between women and men?* - *What should be the role of physicians who decide whether someone can benefit from reproductive technology treatments and start a family?* - In your opinion, does society think differently about parents (especially older parents) who have to resort to reproductive technology (RT) to have children (compared to people who became parents with a spontaneous pregnancy earlier in their lives)?  *Follow-up question: Do you think that there are general differences in how society thinks about how someone gets pregnant (excluding age)?* - In your opinion, what could society / politics do to better address the needs of couples who wish to have children and become parents at an advanced age? |
| Concept of the family / parenthood | - How would you define the term “good (enough)” in relation to parenting / being a parent?  *Follow-up question: Does being older affect the meaning of «good» in relation to «being a parent» in any way? – Or are other factors more determining and consequently “more important”?* - For my next question, I will use this timeline (see end of interview guide). On this timeline you can see different arrows: “too early to have children”, “quite early for children but acceptable”, “ideal time for children”, “a little late for children but acceptable” and “too late to have children”. As you can see, all of the arrows are the same lengths, however, this does not necessarily mean that the time spans must also be the same length. I would very much like to know what you think about this.  So my first question is: When is it too early, quite early, quite late or too late for children? Can you give a specific age? It doesn’t have to be exact, but it can be a period of time, for example five or ten years. (+Can you give any reasons for your statements?)  *Follow-up question:* - *Are there differences between men and women?* - *Are there any advantages or disadvantages for (a) parents; (b) their children; (c) the family as a whole?* |
| Conclusion | Thank you very much. We have now discussed a number of things. Is there anything that you would like to mention or add that has not come up in the conversation so far, but that you think is important? Thank you very much for your participation. |
| Timeline exercise |  |

**Interview guide aspiring parents**

| **Part of the interview guide** | **Interview questions** |
| --- | --- |
| Potential follow-up questions/questions to keep the conversation going or to go into more depth:   - What does that mean exactly? Can you describe this in more detail? - Do you have an example so I can imagine this more concretely? - I don’t fully understand can you explain this a little more? - How was that for you? How did you feel at the time / what were you thinking? - Is there anything else? What else? And further? And then? - What do you mean by that? How do you mean that? How did it continue? | |
| Experiences in relation to reproductive technologies | - Before we begin, it would be nice if you could tell me a little bit about your family. That way I know who we are talking about during the interview and can follow your stories a little easier. - Could you tell me about the moment when you and your partner started thinking about having children? How was this? What were your thoughts and feelings about starting a family? - Could you tell me a little more about your attempts to get pregnant when you first developed a desire to have children?  *Follow-up questions:* - *How did you feel when you realized that it did not work out right away with getting pregnant?* - *Did you search for support or advice (from friends, family, doctors, etc.)?* - If you have already definitely decided for or against one of the existing reproductive technology treatment options: How did the decision-making process take place?  *Follow-up questions:* - *Have you talked to your partner, your family, your friends, your doctors?* - *Have you considered alternatives to these reproductive technology options (e.g. adoption)?* - *If yes: Was there anything about these alternatives that put you off, causing you to decide against them?* |
| Search for treatment and expectations about the course of treatment  +  Views about parenthood | - How did you experience the search for treatment? - Had you imagined the search for a suitable treatment in advance as it was? Or were there unforeseen difficulties in the process? - Do you have the impression that your age has made it more difficult for you to access an appropriate treatment option? - Do you have the impression that your age will affect your treatment in any way? - At the time you started thinking about having a child, what did it mean to you to become a parent? - What does it mean to you now? - How did your family and your social environment (e.g. friends, work colleagues, etc.) react when they found out about your child wish? - If we now take a look into the future, how do you envision parenthood? - Do you have the impression that your own age will have any specific influence in relation to being a parent?   *Follow-up questions:*   - *Do you have the impression that there will be any advantages or disadvantages in terms of being a parent at your age?* - *What about advantages and disadvantages specific to (a) you as parents; (b) your future children; (c) the family as a whole?* - *If we now dare to take another look into the future: Would you describe yourself as a parent of advanced age?* - *At what age would you generally describe someone as a «parent of advanced age»?* - *In your opinion, what are generally the advantages and disadvantages to parenting at an advanced parental age?* - For the next question, let’s dare to take another look into the future: Do you think that your age will have an influence on your relationship with your child / children?  *Follow-up question:* - *Do you think that your relationship with your child or children will develop differently than if you had become parents earlier? In what way?* - Do you think society will see you differently than people who became parents earlier in their lives?  *Follow-up questions:* - *What do you think how this will show itself?* - *What do you think will be your reaction and how do you think you will feel about it?* |
| Moral reasoning to gain a new understanding of advanced parental age as a basis for better and more appropriate policies and recommend-ations | - Now let’s take a step back. Could you briefly explain to me why you want to become a parent specifically at your age? What were / are the main factors that influenced this decision? - Did society, culture and/or the health care system (or other external factors) play a role in you “only” wanting to become a parent now? - In your opinion, what do you think is the influence of these external factors on couples who become parents at an advanced age?  *Follow-up questions:* - *What should be the role of physicians who decide whether someone can benefit from reproductive technology treatments and start a family?* - *Are there differences between women and men?*      - Generally speaking, what is your opinion about couples who have children only at an advanced age?  *Follow-up question: Are there any advantages and/or disadvantages for (a) parents; (b) children; (c) the family as a whole?* - In your opinion, does society think differently about parents (especially older parents) who have to resort to reproductive technology to have children (compared to people who became parents with a spontaneous pregnancy earlier in their lives)?  *Follow-up question: Do you think that there are general differences in how society thinks about how someone gets pregnant (excluding age)?* - In your opinion, what could society / politics do to better address the needs of couples who wish to have children and become parents at an advanced age? |
| Concept of the family / parenthood | - How would you define the term “good (enough)” in relation to parenting / being a parent?  *Follow-up question: Does being older affect the meaning of «good» in relation to «being a parent» in any way?* - For my next question, I will use this timeline (see end of table). On this timeline you can see different arrows: “too early to have children”, “quite early for children but acceptable”, “ideal time for children”, “a little late for children but acceptable” and “too late to have children”. As you can see, all of the arrows are the same lengths, however, this does not necessarily mean that the time spans must also be the same length. I would very much like to know what you think about this.  So my first question is: When is it too early, quite early, quite late or too late for children? Can you give a specific age? It doesn’t have to be exact, but it can be a period of time, for example five or ten years. (+Can you give any reasons for your statements?)  *Follow-up question: Are there differences between men and women?* |
| Conclusion | Thank you very much. We have now discussed a number of things. Is there anything that you would like to mention or add that has not come up in the conversation so far, but that you think is important? Thank you very much for your participation. |
| Timeline exercise |  |
